# Evaluation of anti-nuclear antibody patterns and their associations with autoimmune disease biomarkers

**DOI:** 10.3389/fimmu.2026.1959587

**Published:** 2026-10-23

**Authors:** Min Kyung Lee, Vincent Ricchiuti

**Affiliations:** 1 Labcorp, Durham, NC, United States; 2 Labcorp North-Central Division, Dublin, OH, United States

**Keywords:** antinuclear antibodies (ANA), autoantibodies, DFS70 antibodies, dsDNA antibodies, extractable nuclear antibodies, HEp-2 cell, lupus (SLE), systemic autoimmune rheumatic diseases (SARD)

## Abstract

**Objective:**

Anti-nuclear antibody (ANA) testing is widely used in the evaluation of systemic autoimmune rheumatic diseases (SARD). We evaluated ANA testing performed at Labcorp between 2015 and 2024 and examined serological associations between ANA titers and HEp-2 indirect immunofluorescence assay (IFA) patterns and commonly used SARD-associated autoantibodies in a large real-world laboratory population.

**Methods:**

We conducted a retrospective review of de-identified results from 8,807,628 ANA tests performed at Labcorp between 2015 and 2024. The primary unit of analysis was the ANA test/specimen; repeated testing from the same individual may be represented. ANA positivity was assessed by IFA at titers of 1:40, 1:80, 1:160, 1:320, 1:640, and 1:1280. Reference-laboratory tests used a positivity threshold of >=1:80, whereas the rheumatology-specialty laboratory used >=1:40. Among ANA-positive specimens, 696,456 underwent testing for one or more SARD-associated autoantibodies using routine reflex or ordered testing panels.

**Results:**

ANA positivity decreased progressively with increasing titer thresholds, and positivity rates differed substantially between the reference and rheumatology-specialty laboratory settings. Speckled and homogeneous patterns were the most frequent patterns. Centromere patterns were generally detected at higher titers and were strongly associated with centromere antibody positivity. Among ANA-positive specimens that underwent additional autoantibody testing, the proportion with one or more positive SARD-associated autoantibodies generally increased with ANA titer. Several less common patterns showed associations with individual autoantibodies; these findings are exploratory and require independent validation.

**Conclusion:**

This large real-world laboratory dataset quantifies relationships among ANA titer, HEp-2 IFA pattern, and SARD-associated autoantibody positivity. Higher ANA titers were associated with a greater frequency of disease-associated serological markers, but these data do not establish an optimal diagnostic ANA cutoff or a clinical diagnosis of SARD. Common pattern-autoantibody relationships were confirmed at scale, while associations involving uncommon ANA patterns and anti-DFS70 should be interpreted cautiously and validated in clinically characterized cohorts.

## Introduction

The presence of anti-nuclear antibodies (ANA) is a hallmark for many systemic autoimmune rheumatic diseases (SARD) like systemic lupus erythematosus (SLE), Sjögren’s syndrome (SjS), systemic sclerosis (SSc), mixed connective tissue disease (MCTD), and idiopathic inflammatory myopathies (IIM) (1, 2). As such, ANA is used for or supports SARD diagnosis like SLE, SSc, and SjS (1, 3–5). ANA is categorized into the following patterns: homogeneous, centromere, speckled, nuclear dot, nucleolar, nuclear envelope, anti-proliferating cell nuclear antigen (PCNA), among others (6–8). Specific patterns can clinically be associated with certain SARDs. For example, homogeneous ANA patterns are found in patients with SLE, chronic autoimmune hepatitis, or juvenile idiopathic arthritis (7, 9). Centromere ANA patterns are commonly found in patients with limited cutaneous SSc (9).

While most ANA patterns can occur in SARD, the dense fine speckled (DFS) pattern and anti-DFS70 antibodies have also been reported in ANA-positive individuals without SARD. DFS70 (also known as LEDGF/p75, lens epithelium-derived growth factor, 75 kDa isoform) is a nuclear protein encoded by the PSIP1 gene and has roles in transcriptional regulation, cellular stress responses, and viral integration (10, 11). Anti-DFS70 antibodies are relatively common in the general population and are often associated with a DFS ANA pattern (12, 13). Their isolated presence has been proposed as a serological finding associated with a lower likelihood of SARD in selected populations (12–17). However, anti-DFS70 should not be interpreted as a stand-alone exclusionary marker and requires correlation with other disease-associated autoantibodies and clinical findings.

Specimens with positive ANA results often are reflexed to various tests for biomarkers associated with SARD to distinguish among the various specific diseases within SARD. SARD-associated biomarkers include centromere, chromatin, dsDNA, SS-A, SS-B, RNP, among others. For example, it is recommended to follow up with anti-dsDNA tests and/or with chromatin tests if the patient is suspected of having SLE (9). Scl-70 tests are recommended if SSc is suspected (17). Furthermore, SS-A and SS-B are detected in 50 - 70% of patients with SjS and 10 - 30% in SLE (18–21).

As a national reference laboratory, Labcorp performs substantial volumes of ANA testing across the United States each year (22). In this study, we evaluated ANA testing performed at Labcorp from 2015 to 2024 to quantify real-world serological relationships among ANA titers, HEp-2 IFA patterns, and commonly used SARD-associated autoantibodies. The scale of the dataset also permits quantitative characterization of uncommon ANA patterns that are difficult to evaluate in smaller cohorts.

## Materials and methods

### Study cohort and assays

We conducted a retrospective review of de-identified results from 8,807,628 ANA tests performed at Labcorp from January 2015 through December 2024. The primary unit of analysis was the ANA test/specimen rather than a confirmed unique patient. Because the dataset was de-identified and encompassed a 10-year period, repeated testing from the same individual may be represented; therefore, test-level frequencies should not be interpreted as patient-level disease prevalence estimates. The study included 8,443,953 tests performed through the Labcorp reference laboratory and 363,675 tests performed through RDL, a specialized rheumatology laboratory (**Supplementary Table 1**). For reference-laboratory testing using an automated ANA by IFA system, titers of 1:80 or greater were considered ANA-positive. For rheumatology-specialty laboratory testing using the same methodology, titers of 1:40 or greater were considered ANA-positive (HEp-20–10 cells, EUROPattern, Euroimmun, Lubeck, Germany) (22).

Providers who order ANA tests through the reference laboratory are 30.2% hospital laboratory providers, 27.3% primary care providers, and 12.9% rheumatology providers. Providers who order ANA tests through the rheumatology laboratory are 32.8% rheumatology providers, 31.2% primary care providers, and 6.3% hospital laboratory providers (**Supplementary Figure 1A**).

ANA-positive specimens were characterized by reported IFA patterns, including speckled, homogeneous, centromere, nuclear dot, spindle apparatus, midbody, nuclear membrane, centriole, and PCNA. Among ANA-positive specimens, 696,456 underwent testing for one or more SARD-associated autoantibodies, including centromere, chromatin, dsDNA, Jo-1, ribosomal P, SS-A, SS-B, Scl-70, Sm, Sm/RNP, RNP, and U1 RNP. Autoantibody testing was not uniform across all specimens because routine clinical orders and reflex algorithms included different biomarker combinations; therefore, denominators vary by biomarker and an unmeasured biomarker was not considered negative. Testing methods included addressable laser bead immunoassay (ALBIA) (BioPlex 2200, Bio-Rad, Hercules, California, USA) and enzyme-linked immunosorbent assays (QuantaLite, Werfen, San Diego, California, USA). dsDNA testing included indirect fluorescent antibody using Crithidia luciliae (EUROPattern, Euroimmun, Lubeck, Germany), the Fluorescent Anti-double-stranded DNA Antibody Ratio (FAAR) method, and ALBIA.

Dense fine speckled (DFS) patterns are currently reported as comments for ANA testing performed through the reference laboratory. We evaluated anti-DFS70 results from 161 specimens with a DFS pattern noted in the ANA result. Anti-DFS70 levels greater than 20 units were considered positive (QuantaLite, Werfen, San Diego, California, USA). We also evaluated 3,382 anti-DFS70 tests from specimens with corresponding ANA testing. Because anti-DFS70 testing was performed in a selected subgroup rather than an unbiased population, these analyses were considered exploratory and were not used to estimate the diagnostic performance of anti-DFS70 for SARD.

The geographic location of each subject was grouped by regions defined by the United States Census. The Northeast region includes Connecticut, Maine, Massachusetts, New Hampshire, Rhode Island, Vermont, New Jersey, New York, and Pennsylvania. The Midwest region includes Illinois, Indiana, Michigan, Ohio, Wisconsin, Iowa, Kansas, Minnesota, Missouri, Nebraska, North Dakota, and South Dakota. The South region includes Delaware, District of Columbia, Florida, Georgia, Maryland, North Carolina, South Carolina, Virginia, West Virginia, Alabama, Kentucky, Mississippi, Tennessee, Arkansas, Louisiana, Oklahoma, and Texas. The West region includes Arizona, Colorado, Idaho, Montana, Nevada, New Mexico, Utah, Wyoming, Alaska, California, Hawaii, Oregon, and Washington.

### Statistical analysis

Comparisons of proportions for categorical variables (including rheumatology-associated ICD-10 codes) and comparisons of SARD-associated biomarker positivity rates were performed using two-sample proportion Z-tests. Associations between specific ANA patterns and SARD-associated autoantibody positivity were evaluated using multivariable logistic regression models adjusted for patient sex, age, laboratory setting, and ANA pattern titerTo increase confidence in pattern assignment for these association analyses, only patterns identified at ANA titers of 1:320 or greater were included. P values less than 0.001 were reported as p < 0.001. Where multiple comparisons were performed and indicated in the corresponding analysis, P values were adjusted using the Benjamini-Hochberg method. Given the very large sample size, effect sizes and 95% confidence intervals were considered together with P values, and findings involving uncommon patterns were treated as exploratory.

### Funding and patients consent

The study was conducted in accordance with the ethical principles of the Declaration of Helsinki, adopted by the 18th World Medical Assembly in Helsinki, Finland, in June 1964. Samples were blinded before analysis to protect confidentiality. The Western Institutional Review Board® (Puyallup, WA, USA) determined the study protocol to be exempt from review.

## Results

**Table 1** summarizes demographic characteristics, regional distribution, ANA titers, ANA patterns, and SARD-associated biomarker testing among ANA-positive and ANA-negative tests/specimens from the reference and rheumatology-specialty laboratories between 2015 and 2024. The two settings differed substantially in patient selection, ANA positivity thresholds, provider mix, and use of reflex biomarker testing. These differences are important when comparing positivity rates and serological profiles across the two laboratory populations.

**Table 1:** Specimen characteristics by ANA positivity and laboratory the test was performed at.

Characteristic	ANA-positive		ANA-negative	
	Reference	Rheumatology	Reference	Rheumatology
**N (% of ANA tests from laboratory type)**	2036314 (24.1)	318890 (86.7)	6407248 (76.9)	48796 (13.3)
**Age, median [Q1, Q3]**	54 [40, 66]	52 [38, 65]	53 [39, 65]	53 [38, 65]
Sex, N (%)				
Female	1617412 (79.4)	247379 (77.6)	4277358 (66.8)	34128 (69.9)
Male	417987 (20.5)	71312 (22.4)	2126884 (33.2)	14627 (30)
Unknown	915 (0)	199 (0.1)	3006 (0)	41 (0.1)
Region, N (%)				
Midwest	287681 (14.1)	19177 (6.0)	603241 (9.4)	2996 (6.1)
Northeast	572749 (28.1)	46462 (14.6)	1280311 (20)	6712 (13.8)
South	791229 (38.9)	154708 (48.5)	3032167 (47.3)	24859 (50.9)
West	236792 (11.6)	92350 (29.0)	945332 (14.8)	13013 (26.7)
US Territories	7033 (0.3)	138 (0.0)	22465 (0.4)	14 (0)
Unknown	140830 (6.9)	6055 (1.9)	523732 (8.2)	1202 (2.5)
ANA titer, N (%)				
1:40	0 (0.0)	67066 (21)		
1:80	727266 (35.7)	60706 (19)		
1:160	531499 (26.1)	103336 (32.4)		
1:320	339164 (16.7)	38626 (12.1)		
1:640	204406 (10)	23631 (7.4)		
1:1280	143795 (7.1)	8252 (2.6)		
>1:1280	90184 (4.4)	17273 (5.4)	8811248	
# of ANA patterns present, N (%)				
1	1810180 (88.9)	239023 (75)		
2	220011 (10.8)	73145 (22.9)		
3+	6123 (0.3)	6722 (2.1)		
**Has SARD-associated biomarker testing, N (%)**	426276 (20.9)	273606 (85.8)		

The ANA positivity rate was substantially higher in the rheumatology-specialty laboratory than in the reference laboratory. This difference should not be interpreted as an effect of laboratory setting itself. The rheumatology-specialty laboratory serves a more selected population with higher pretest probability of autoimmune disease, and it uses a lower ANA positivity threshold (1:40 versus 1:80 in the reference laboratory). Both factors contribute to the observed difference in positivity rates (**Supplementary Table 1**).

Demographic features of ANA-positive specimens were broadly similar across settings. The median ages associated with ANA-positive tests were 54 years in the reference laboratory and 52 years in the rheumatology-specialty laboratory, and most ANA-positive tests were from female patients (79.4% and 77.6%, respectively).

Regional distributions also differed between settings. In the reference laboratory, the largest proportions of ANA-positive tests were from the South (38.9%) and Northeast (28.1%). In the rheumatology-specialty laboratory, the highest representation was from the South (48.5%) and West (29.0%). These differences may reflect referral patterns, geographic variation in clinical practice, and access to specialty testing.

The distribution of ANA titers further illustrates differences between the testing populations. In the reference laboratory, most positive tests were at lower titers (1:80 in 35.7% and 1:160 in 26.1%), whereas higher titers (>=1:640) were less frequent (21.5% combined). In the rheumatology-specialty laboratory, the most frequent titer was 1:160 (32.4%), with positive tests also represented at both the 1:40 threshold (21.0%) and >1:1280 (5.4%). These differences may reflect patient selection, referral patterns, pretest probability, and laboratory-specific reporting thresholds.

Differences were also observed in the number of ANA patterns reported. A single ANA pattern predominated in both settings but was more frequent in the reference laboratory (88.9% versus 75.0%). Multiple patterns (>=2) were reported more often in the rheumatology-specialty laboratory (25.0% versus 11.1%). These differences should be interpreted in the context of the distinct testing populations and laboratory workflows.

SARD-associated biomarker testing was more common in the rheumatology-specialty laboratory (85.8%) than in the reference laboratory (20.9%), consistent with more extensive serological evaluation in a specialty setting. Because biomarker panels and reflex algorithms differed, subsequent biomarker analyses are conditional on the individual biomarker having been tested.

Rheumatology-associated ICD-10 codes were submitted with a higher proportion of ANA-positive tests performed through the rheumatology-specialty laboratory than through the reference laboratory (37.4% versus 25.1%, p < 0.001; **Supplementary Figure 1B**). Similarly, a higher proportion of ANA-positive tests in the rheumatology-specialty laboratory were ordered by rheumatology providers (35.9% versus 20.3%, p < 0.001; **Supplementary Figure 1C**). These variables characterize the clinical context of testing but do not constitute adjudicated SARD diagnoses.

Together, these findings demonstrate substantial differences between the two real-world testing settings. Reference-laboratory data reflect a broad clinical testing population, whereas rheumatology-specialty laboratory data reflect more selected specialty testing. Differences in pretest probability, ANA cutoff, provider mix, and reflex testing should therefore be considered when interpreting comparisons between settings.

### ANA patterns and titers

We first evaluated the ANA positivity at different titer cutoffs. As expected, positivity decreased with each increasing titer threshold (**Figure 1A**). As tests performed at reference laboratories do not report ANA positive results at the 1:40 titer, positivity at ≥1:40 were only considered for ANA tests performed at rheumatology specialty laboratories (**Supplementary Table 1**). 24.1% of ANA tests performed at reference laboratories were positive at the 1:80 threshold, 15.5% were positive at the 1:160 threshold, 9.2% were positive at the 1:320 threshold, 5.2% were positive at the 1:640 threshold, and 2.8% were positive at the 1:1280 threshold (**Figure 1A**). 87.7% of ANA tests performed at rheumatology specialty laboratories were positive at the 1:40 threshold, 69.2% were positive at the 1:80 threshold, 52.6% were positive at the 1:160 threshold, 24.1% were positive at the 1:320 threshold, 13.5% were positive at the 1:640 threshold, and 7.0% were positive at the 1:1280 threshold (**Figure 1A**).

**Figure 1:**
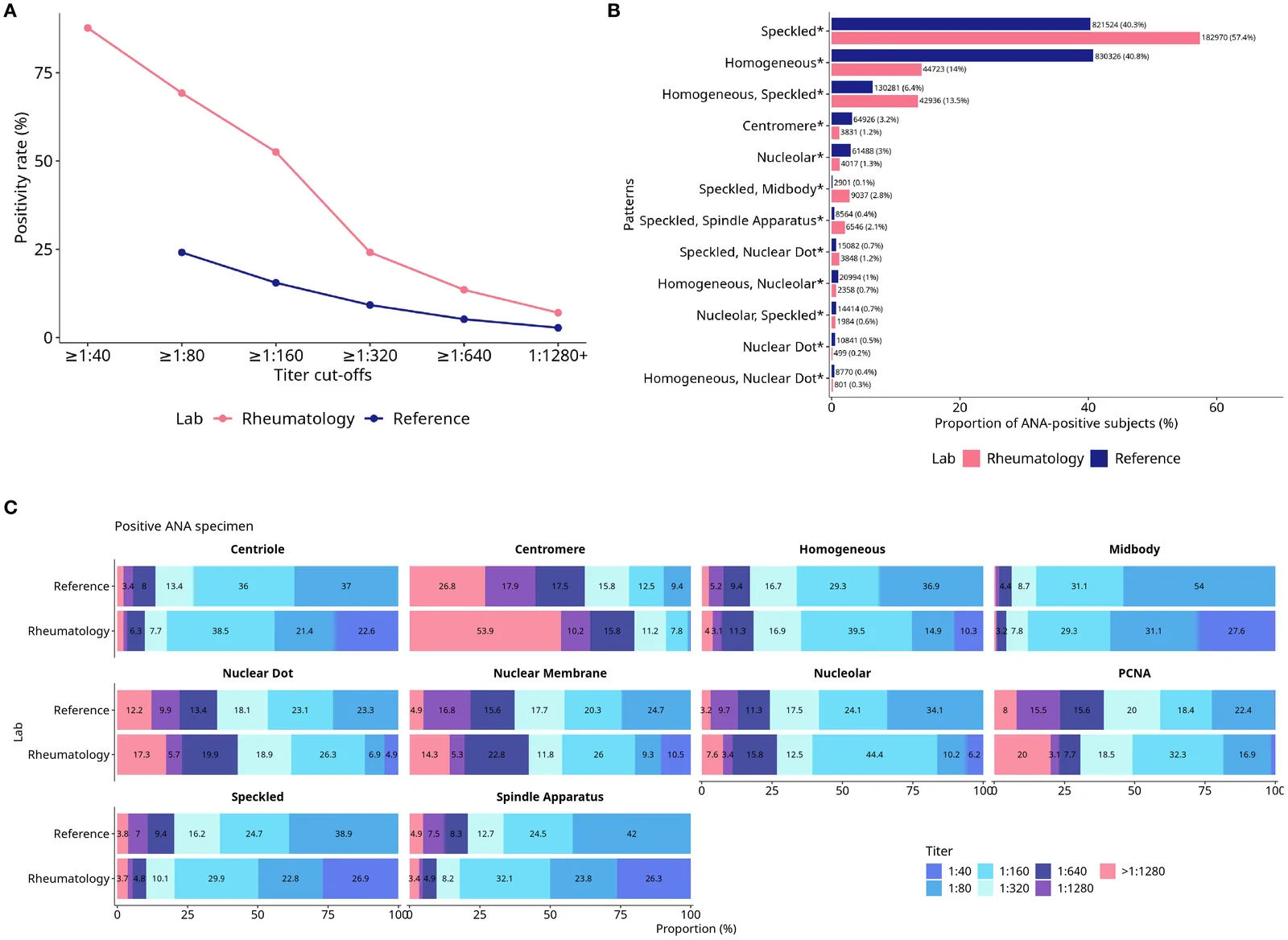
ANA positivity and patterns. **(A)** ANA positivity rates at various titer cut-offs grouped by the laboratory (reference or rheumatology specialty labs) the ANA test was performed at. **(B)** Proportions of ANA pattern(s) in ANA-positive specimen based on which lab the ANA test was performed at. Comparisons of proportions between the labs were conducted with two-sample proportional Z-tests. *: p < 0.001. **(C)** Distributions of titers for each ANA pattern by the laboratory the ANA test was performed at.

The two most common ANA patterns in ANA-positive specimens were speckled and homogeneous patterns in ANA tests performed at reference laboratories and rheumatology specialty laboratories (**Figure 1B**; **Supplementary Table 2**). 87.2% of ANA-positive specimen had homogeneous, speckled, or homogeneous and speckled patterns regardless of laboratory the tests were performed at. However, speckled patterns were present at higher proportions in ANA-positive specimens with tests performed at rheumatology specialty laboratories (57.4%) compared to ANA-positive specimens with tests performed at reference laboratories (42.6%, p < 0.001). Homogeneous patterns were present at higher proportions in ANA-positive specimens with tests performed at reference laboratories (40.8%) compared to ANA-positive specimens with tests performed at rheumatology specialty laboratories (14.0%, p < 0.001). Other ANA patterns were identified at lower prevalences. 3.2% of ANA-positive specimens in tests performed at reference laboratories had centromere patterns and 3.0% had nucleolar patterns (**Figure 2B**). 2.8% of ANA-positive specimens in tests performed at rheumatology specialty laboratories had midbody with speckled patterns, and 2.1% had spindle apparatus with speckled patterns (**Figure 2B**).

**Figure 2:**
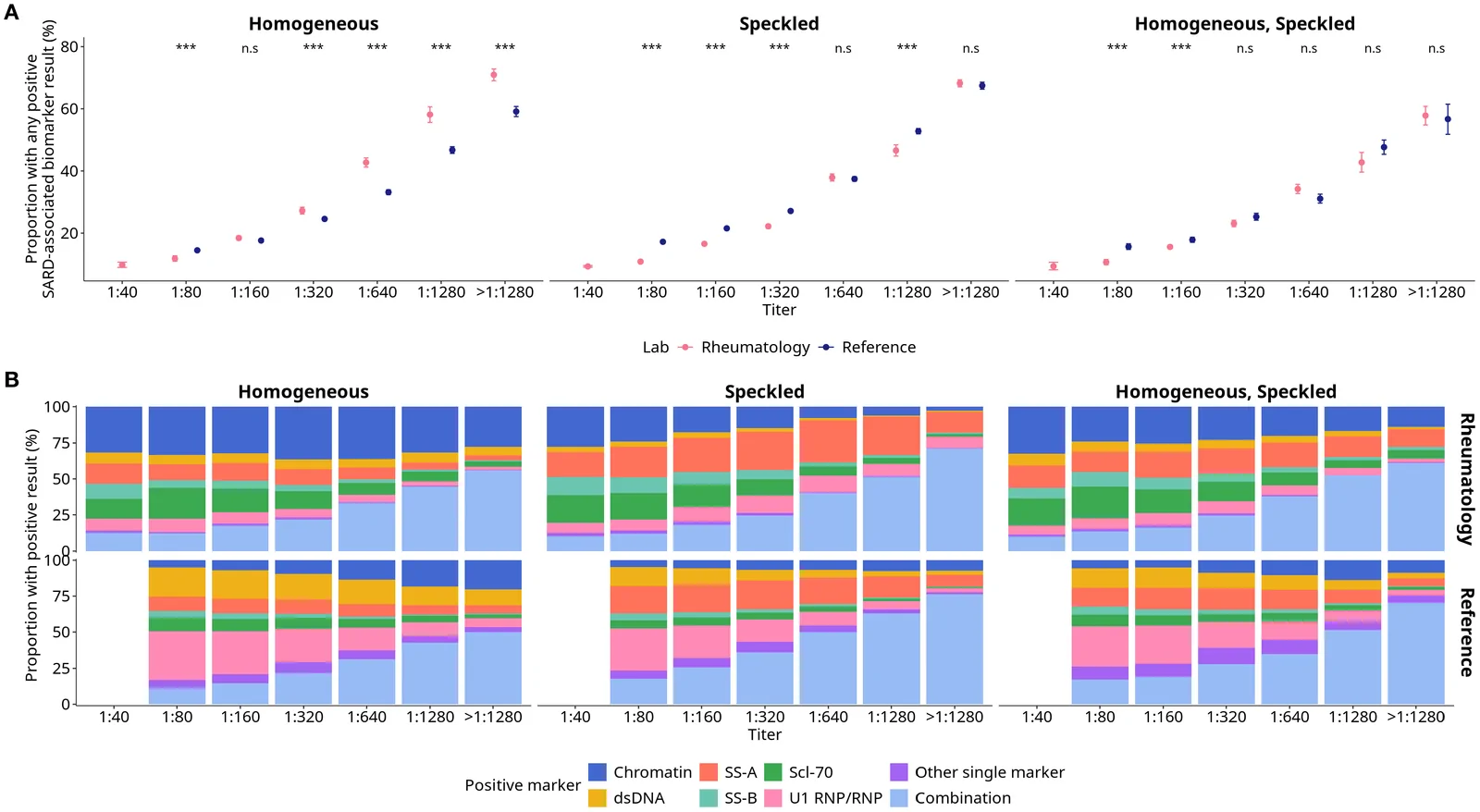
SARD-associated biomarkers in ANA-positive specimen with homogeneous, speckled, or homogeneous and speckled patterns. **(A)** Proportion of specimen with positivity for any SARD-associated biomarker at the titer of ANA positivity. Error bars indicate lower and upper bounds of the 95% confidence interval of the proportions. Comparisons of proportions between the labs were conducted with two-sample proportional Z-tests. ***: p-value < 0.001. n.s: not significant. **(B)** Distribution of positive markers among specimens with any positive SARD-associated biomarker at each ANA titer. Other single markers include markers such as centromere, Jo-1, ribosomal P, smith/RNP, and smith.

We investigated the titers at which each pattern was found to better understand ANA patterns. More common patterns like homogeneous or speckled patterns were identified at lower titers. For example, 66.2% of specimens with homogeneous patterns were identified with 1:80 and 1:160 titers in ANA tests performed with reference laboratories and 64.7% of specimens with homogeneous patterns were identified with 1:40, 1:80, and 1:160 titers in ANA tests performed with rheumatology specialty laboratories (**Figure 1C**). However, less common patterns like centromere or PCNA patterns were identified at higher titers. For example, 44.7% and 64.1% of specimens with centromere patterns in ANA tests performed at reference laboratories and rheumatology specialty laboratories, respectively, were identified at 1:1280 or greater titers (**Figure 1C**).

### Associations between ANA patterns and SARD-associated biomarkers

Among ANA-positive specimens with additional SARD-associated biomarker testing, the combinations of biomarkers measured varied substantially. The most common panel, performed in 34.7% of specimens with reflex testing, included centromere, chromatin, Jo-1, RNP, SS-A, SS-B, Scl-70, Smith, and dsDNA; another common panel, performed in 28.8%, included centromere, chromatin, SS-A, SS-B, Scl-70, Smith, U1 RNP, and dsDNA (**Supplementary Figures 2A, B**; **Supplementary Table 3**). Because an unmeasured biomarker cannot be assumed to be negative, analyses of individual biomarkers used the specimens in which that biomarker was actually tested. Positivity for at least one tested SARD-associated biomarker generally increased with ANA titer for homogeneous, speckled, and combined homogeneous/speckled patterns (**Figure 2A**), although the magnitude differed between laboratory settings.

We investigated two groups of ANA-positive specimens with the two most common panel of reflexed biomarkers. Among the ANA-positive specimens with reflex tests for centromere, chromatin, jo-1, RNP, SS-A, SS-B, Scl-70, Smith, and dsDNA, 26.7% had at least one positive biomarker. Among those with at least one positive biomarker, 70.2% were positive for only one biomarker (**Supplementary Figure 3A**). Similar patterns were found among the ANA-positive specimens with reflex tests for centromere, chromatin, SS-A, SS-B, Scl-70, Smith, U1 RNP, and dsDNA. 20.3% of these specimens were positive for at least one biomarker and among those, 73.8% were positive for only one biomarker (**Supplementary Figure 3B**).

Increasing proportions of ANA-positive specimen with homogeneous, speckled, or homogeneous and speckled patterns had multiple positive SARD-associated biomarkers with increasing titer (**Figure 2B**). In specimens with homogeneous patterns with tests performed at rheumatology specialty laboratories, 27.9 – 36.0% of the specimens were positive for just chromatin across the titers. In specimens with speckled patterns with tests performed at rheumatology specialty laboratories, 14.8 – 29.1% of the specimens were positive for just SS-A across the titers. In specimens with homogeneous patterns with tests performed at reference laboratories, 11.4 – 20.4% of the specimens were positive for just dsDNA across the titers. In specimens with speckled patterns with tests performed at reference laboratories, 8.3 – 20.1% of the specimens were positive for just SS-A across the titers.

Almost all ANA-positive tests with centromere patterns, serving as our positive controls, especially those identified at titers of 1:320 or greater, had positive centromere antibody results (**Supplementary Figure 4**). In speckled patterns, RNP, Smith/RNP, SS-A, and SS-B are expected to be present. We identified increasing positivity rate of SARD-associated biomarkers with increased titer sensitivity in speckled patterns particularly (**Supplementary Figure 4**). In titers 1:1280 and >1:1280, there were significant differences in positivity rates in chromatin (p < 0.001), dsDNA (p < 0.001), RNP (p < 0.001), Scl-70 (p < 0.001), smith (p < 0.001), smith/RNP (p < 0.001), SS-A (p < 0.001), SS-B (p < 0.001), and U1 RNP (p < 0.001) biomarkers between homogenous and speckled patterns (**Supplementary Figure 4**).

We next evaluated associations between ANA patterns and SARD-associated autoantibody positivity, restricting these analyses to patterns observed at titers of 1:320 or greater with multivariable logistic regression, adjusting for patient sex, age, laboratory setting, and titer of the ANA pattern. Speckled patterns were associated with increased odds of positivity for multiple autoantibodies, with the largest odds ratios observed for SS-B (OR = 4.3, 95% CI = 4.1 – 4.5) and SS-A (OR = 3.2, 95% CI = 3.1 – 3.3). Homogeneous patterns were most strongly associated with dsDNA (OR = 2.5, 95% CI = 2.4 – 2.6) and chromatin (OR = 1.7, 95% CI = 1.7 – 1.7), while centromere patterns showed a very strong association with centromere antibody positivity (OR = 2052.5, 95% CI = 1847.6 – 2280.0) (**Figure 3**; **Supplementary Table 4**). Couple uncommon patterns also showed statistical associations with individual autoantibodies, including spindle apparatus with chromatin, and PCNA with Jo-1 and ribosomal P. Because these rare-pattern analyses were unadjusted, involved smaller denominators, and lack external clinical validation, they should be considered exploratory rather than established clinical relationships.

**Figure 3:**
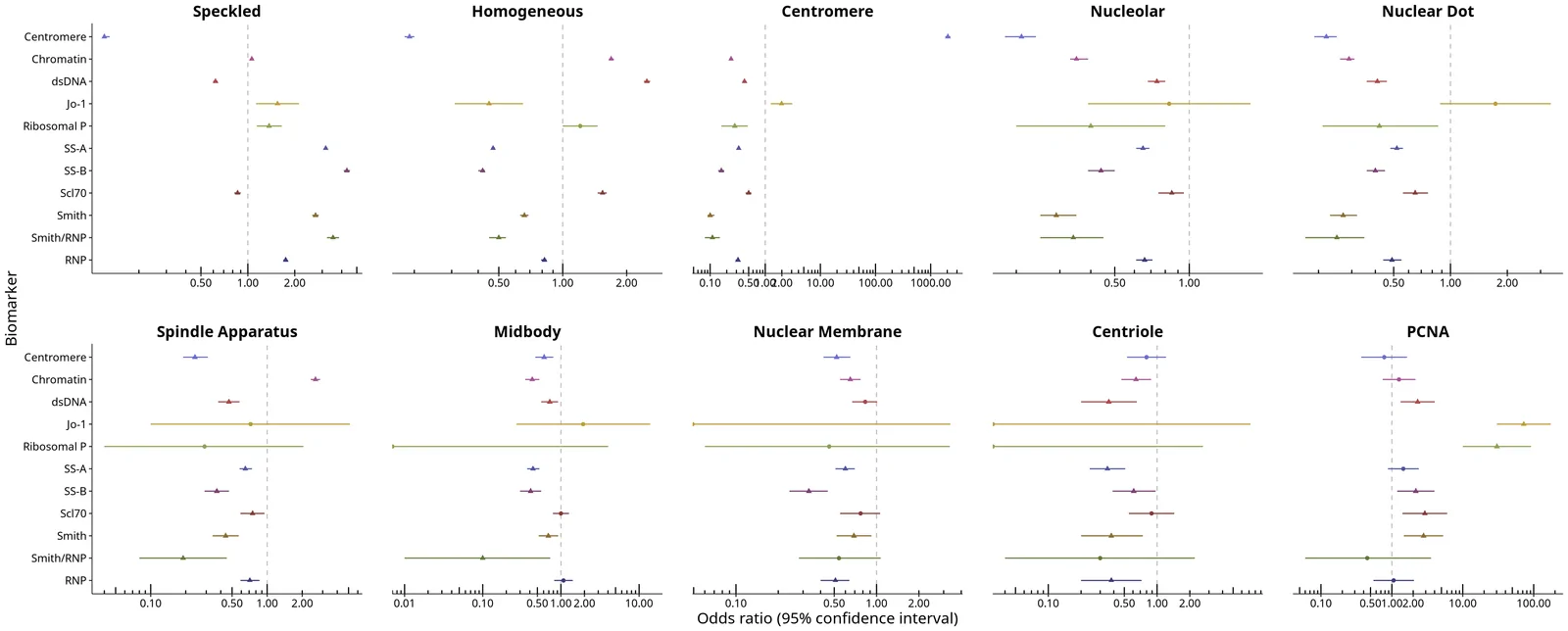
Associations between ANA patterns and SARD-associated biomarkers positivity. Points indicate the odds ratio. Error bars indicated lower and upper bounds of 95% confidence intervals. Odds ratio determined by Fisher’s exact tests. Some patterns did not have enough tests for certain biomarker tests to perform Fisher’s exact test (i.e. Jo-1 and Ribosomal P in midbody patterns).

### Anti-DFS70 antibodies in ANA-positive specimens

We then set out to investigate prevalences of anti-DFS70 positivity among the various ANA patterns as well as its association with SARD-associated biomarkers. First, we identified 98.1% of specimens (N = 161) with dense fine speckled patterns noted in their ANA results had positive results for anti-DFS70 antibodies.

Next, we evaluated anti-DFS70 positivity among 1,702 ANA-positive and 1,626 ANA-negative specimens with anti-DFS70 testing (**Supplementary Table 3**). Anti-DFS70 was positive in 6.6% of ANA-negative specimens (**Figure 4A**). Among ANA-positive specimens, anti-DFS70 positivity was 42.7% for combined homogeneous/speckled patterns, 40.7% for speckled/nuclear-dot patterns, 30.1% for speckled patterns, and 25.5% for homogeneous patterns. In specimens with speckled patterns, anti-DFS70 positivity was higher at titers of 1:320 (55.6%), 1:640 (45.4%), and 1:1280 (42.3%) than at 1:80 (22.4%), with adjusted p values as shown in **Supplementary Figure 5**. These estimates apply to the selected subgroup in which anti-DFS70 testing was performed and should not be interpreted as population prevalence.

**Figure 4:**
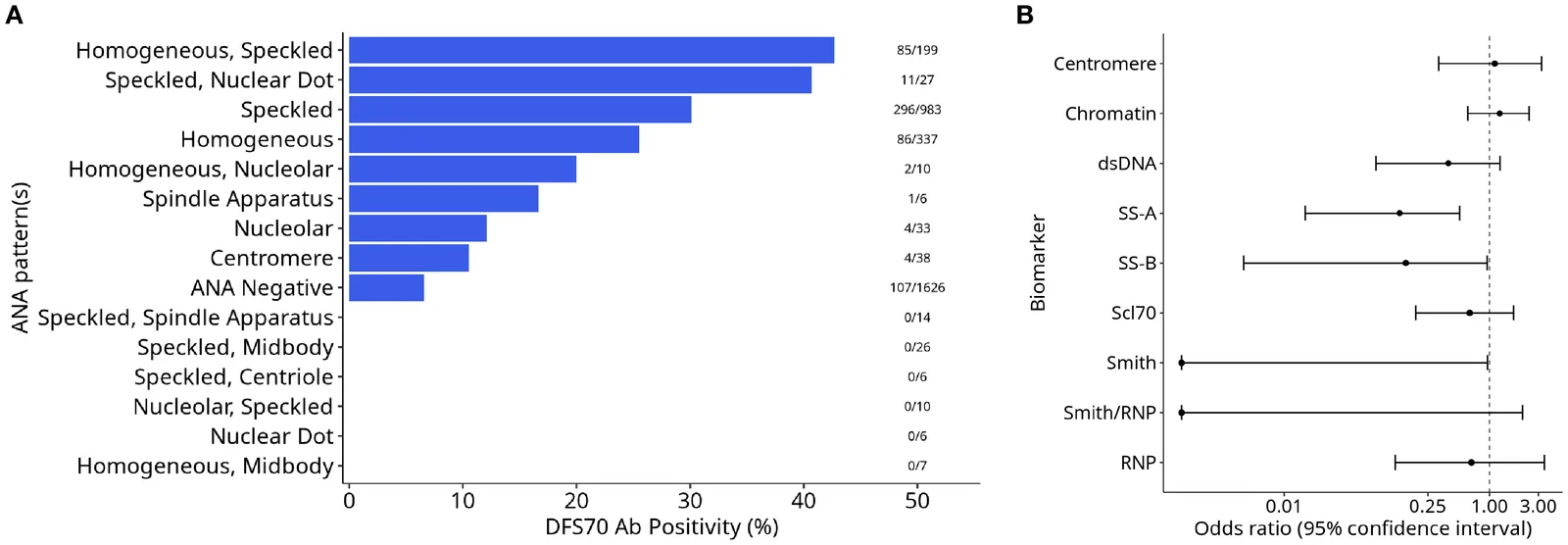
Anti-DFS70 antibody in various ANA patterns and its associations with other SARD-associated biomarkers. **(A)** Anti-DFS70 antibody (Ab) positivity rate in various ANA patterns. The number of specimens with positive anti-DFS70 antibodies and total number of specimens for each ANA pattern with anti-DFS70 testing are indicated on the right side of the bar as N/N. Only patterns with at least 5 anti-DFS70 antibody tests were included. **(B)** Associations between anti-DFS70 antibody positivity and positivity in SARD-associated biomarkers. Points indicate the odds ratio. Error bars indicated lower and upper bounds of 95% confidence intervals. Odds ratio determined by Fisher’s exact tests. Some patterns did not have enough tests for certain biomarker tests to perform Fisher’s exact test (i.e. Jo-1 and ribosomal P).

Because isolated anti-DFS70 has been reported to occur more often in individuals without SARD, we evaluated its serological relationship with other SARD-associated autoantibodies. Anti-DFS70 positivity was negatively associated with SS-A (OR = 0.1, 95% CI = 0.02-0.5), SS-B (OR = 0.2, 95% CI = 0.004-0.95), and Smith antibodies (OR = 0.00, 95% CI = 0.00-0.95; **Figure 4B**), and was not significantly associated with several other tested autoantibodies. Because anti-DFS70 testing was performed in a selected subgroup and clinically adjudicated SARD diagnoses were unavailable, these data do not establish the sensitivity, specificity, or negative predictive value of anti-DFS70 for excluding SARD.

## Discussion

This study evaluates ANA testing at a scale that is difficult to achieve in clinically adjudicated cohorts. The principal contribution is therefore the large real-world quantification of relationships among ANA titer, HEp-2 IFA pattern, laboratory setting, and SARD-associated autoantibody positivity rather than the discovery of entirely new biological relationships. Across more than 8.8 million ANA tests, homogeneous and speckled patterns predominated, centromere patterns showed a strong relationship with centromere antibody positivity, and higher ANA titers were generally accompanied by a greater frequency of positive disease-associated autoantibodies among specimens that underwent additional testing.

ANA positivity decreased as the titer threshold increased, while the proportion of tested specimens with one or more SARD-associated autoantibodies generally increased with titer. This finding is consistent with the established concept that ANA titer influences the clinical significance of a positive result. However, the present study does not contain systematically adjudicated SARD diagnoses and therefore cannot determine an optimal diagnostic cutoff. In particular, our data should not be interpreted as establishing 1:80 or 1:160 as universally preferable thresholds; selection of a cutoff depends on the clinical context, testing strategy, pretest probability, and the intended balance between sensitivity and specificity.

Several findings confirm well-established pattern-autoantibody relationships at very large scale. Homogeneous patterns showed increased odds of chromatin and dsDNA positivity, consistent with prior descriptions of the clinical relevance of homogeneous HEp-2 IFA patterns, while centromere patterns were strongly associated with centromere antibody positivity. Speckled patterns were associated with a broader range of autoantibodies, including RNP, Sm/Sm-RNP, SS-A, and SS-B. These observations are not presented as novel disease-specific relationships; rather, the large dataset provides quantitative estimates across titers and across two distinct real-world laboratory populations.

The large sample size also permitted evaluation of uncommon ANA patterns. Statistical associations involving PCNA, spindle apparatus, midbody, nuclear membrane, and centriole patterns are potentially informative, but they should be regarded as hypothesis-generating. These patterns were uncommon, individual biomarker denominators varied because of clinical testing practices, and the analyses were not adjusted for important potential confounders. Apparent associations, particularly large odds ratios with wide confidence intervals in rare groups, require replication in independent cohorts with standardized testing and clinical phenotyping before they are assigned diagnostic significance.

Anti-DFS70 findings also require cautious interpretation. In the selected specimens in which anti-DFS70 was tested, positivity showed negative or absent associations with several SARD-associated autoantibodies. This is directionally consistent with prior literature suggesting that isolated anti-DFS70 may be more common in individuals without SARD. Nevertheless, our study did not include systematic clinical adjudication, and anti-DFS70 testing was not performed in an unbiased population. Accordingly, these results do not support using anti-DFS70 as a stand-alone test to rule out SARD; its presence should be interpreted together with the overall ANA pattern, disease-associated autoantibodies, and clinical findings.

The reference and rheumatology-specialty laboratory populations also differed substantially. The specialty population had a lower ANA reporting threshold, greater representation of rheumatology providers and rheumatology-associated ICD-10 codes, and substantially more frequent SARD-associated biomarker testing. These differences indicate distinct pretest probabilities and testing pathways. Comparisons between the settings are therefore descriptive and should not be interpreted as causal effects of laboratory setting.

Several limitations should be emphasized. First, the study evaluates laboratory results rather than clinically adjudicated SARD diagnoses; autoantibody positivity does not establish disease, and clinical false-positive or false-negative rates cannot be calculated. Second, the unit of analysis is the test/specimen, and repeated testing from the same individual may be included. Third, SARD-associated autoantibody testing was not uniform, creating selection/verification bias and biomarker-specific denominators. Fourth, pattern-autoantibody association analyses were unadjusted, so residual confounding by ANA titer, laboratory setting, age, sex, and other factors is possible. Fifth, the large number of comparisons increases the likelihood of statistically significant findings of limited clinical magnitude, particularly among rare patterns. These limitations support interpreting the uncommon-pattern and anti-DFS70 findings as exploratory.

In conclusion, this large national laboratory dataset confirms and quantifies established relationships among ANA titer, HEp-2 IFA patterns, and SARD-associated autoantibodies in real-world testing. Its principal added value is the scale of the dataset, comparison of two different testing settings, and quantitative characterization of uncommon patterns. Future studies linking standardized ANA and autoantibody results to unique-patient longitudinal data and clinically adjudicated SARD diagnoses will be needed to determine diagnostic performance and validate the exploratory associations observed here.

## Data Availability

The original contributions presented in the study are included in the article/**Supplementary Material**. Further inquiries can be directed to the corresponding author.
